# Associations of the A118G OPRM1 polymorphism with sociotropy and interpersonal sensitivity

**DOI:** 10.1002/brb3.2674

**Published:** 2022-06-27

**Authors:** Akihito Suzuki, Toshinori Shirata, Keisuke Noto, Yoshihiko Matsumoto, Haruka Muraosa, Mio Abe, Kaoru Goto, Koichi Otani

**Affiliations:** ^1^ Department of Psychiatry Yamagata University School of Medicine Yamagata Japan; ^2^ Department of Anatomy and Cell Biology Yamagata University School of Medicine Yamagata Japan

**Keywords:** autonomy, interpersonal sensitivity, OPRM1, sociotropy

## Abstract

**Background:**

The μ‐opioid receptor (MOR) plays an important role in social bonding behaviors, while it is implicated in the pathophysiology of depression. It is shown that the A118G polymorphism (rs1799971) of the MOR gene (OPRM1) causes amino‐acid exchange from Asn to Asp, and that this polymorphism is associated with altered mu‐opioid receptor function. Meanwhile, sociotropy/autonomy and interpersonal sensitivity are personality vulnerabilities to depression characterized by distinctive interpersonal styles. The present study tested the hypothesis that the functional A118G OPRM1 polymorphism influences these personality traits.

**Methods:**

The subjects were 402 physically and mentally healthy Japanese volunteers. Sociotropy and autonomy were measured by the Sociotropy‐Autonomy Scale, and interpersonal sensitivity was evaluated by the Interpersonal Sensitivity Measure. The A118G polymorphism of the OPRM1 was determined by the PCR method.

**Results:**

In one factor analysis of covariance, there were differences in scores of sociotropy (uncorrected *p* < .001, corrected *p* < .003) and interpersonal sensitivity (uncorrected *p* = .015, corrected *p* = .045), but not autonomy, among the A/A, A/G, and G/G genotypes. Post hoc LSD tests showed that sociotropy scores were higher in the A/A group than in the A/G (*p* = .029) and G/G (*p* < .001) groups, and higher in the A/G group than in the G/G group (*p* = .004). Interpersonal sensitivity scores were higher in the A/A group than in the A/G (*p* = .023) and G/G (*p* = .009) groups.

**Conclusion:**

This study suggests that the A118G OPRM1 polymorphism is associated with sociotropy and interpersonal sensitivity, interpersonal vulnerabilities to depression.

## Graphical abstract

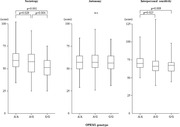

## 1 INTRODUCTION

The endogenous opioid system consists of a family of peptides such as endorphins, enkephalins, and dynorphins, and a family of receptors such as μ (MOR), δ, and κ (Peciña et al., 2019). The A118G single‐nucleotide polymorphism (rs1799971) is located within exon 1 of the OPRM1, the gene encoding MOR, and causes amino‐acid exchange from Asn to Asp at position 40 of MOR (Gelernter et al., 1999). In the cell culture system expressing the OPRM1, the A allele induces higher levels of surface receptor expression, forskolin‐induced cAMP activation, and agonist‐induced receptor activation compared to the G allele (Kroslak et al., 2007). A human autopsy brain study demonstrates that the A allele causes 1.5‐fold higher levels of MOR mRNA and 10‐fold higher levels of MOR protein (Zhang et al., 2005). Positron emission tomography (PET) studies show that baseline MOR availability is higher in subjects with the A allele than in those with the G allele (Peciña et al., 2015; Weerts et al., 2013).

There is extensive evidence that MOR is involved in mammalian bonding behaviors such as mother‐infant attachment, romantic relationships, and affiliative interactions (Machin & Dunbar, 2011). In human adults, low cerebral MOR availability in PET imaging is correlated with avoidant attachment style (Nummenmaa et al., 2015), which is featured by decreased attachment emotions and behaviors (Mikulincer & Shaver, 2007). There is also increasing evidence that altered MOR function is implicated in the pathophysiology of depression. Two PET studies reveal altered MOR availability in several brain areas of depressed patients (Kennedy et al., 2006) and cohort subjects with depressive symptoms (Nummenmaa et al., 2020). Human postmortem brain studies show increased MOR radioligand‐binding density (Gabilondo et al., 1995; Gross‐Isseroff et al., 1990) and high MOR mRNA expression (Escribá et al., 2004) in suicide victims, the majority of them being diagnosed with depression. In genetic association studies, the A allele of the OPRM1 polymorphism predictive of increased activity of MOR (Kroslak et al., 2007; Peciña et al., 2015; Weerts et al., 2013; Zhang et al., 2005) is associated with depressive symptoms in children and adolescents (Adrian et al., 2015; Kleinjan et al., 2015), and completed suicides (Hishimoto et al., 2008). However, the associations of this polymorphism with depression or suicidal behaviors have not been confirmed in a meta‐analysis or a large‐scale genetic association study. There have also been no genome‐wide association studies focusing on the A118G OPRM1 polymorphism or the SNPs around this polymorphism in relation to personality traits or major depression, to our knowledge.

Sociotropy and autonomy are suggested as two types of personality associated with vulnerability to depression in Beck's cognitive model (Beck, 1983). Persons high in sociotropy have self‐schemas referring to interpersonal closeness, acceptance, and approval, and these persons are susceptible to stressors perceived as an interpersonal loss like separation, rejection, and disapproval. On the other hand, persons high in autonomy have self‐schemas referring to accomplishment, independence, and self‐control, and these persons are vulnerable to stressors perceived as a failure to achieve and a loss of independence or self‐control. Longitudinal studies show that sociotropic persons and autonomic persons are actually sensitive to life events congruent with contents of respective self‐schemas, leading to development of depression (Hammen et al., 1989; Morse & Robins, 2005). Meanwhile, interpersonal sensitivity is featured by undue and excessive awareness of, and susceptibility to, behavior and feelings of others (Boyce & Parker, 1989). Persons high in this personality trait are preoccupied with behavior and moods of others, overly reactive to perceived or actual criticism or rejection, and they modify their behavior in order not to be criticized or rejected. A meta‐analysis on longitudinal data shows that interpersonal sensitivity is a vulnerable factor to depression (Gao et al., 2017). Twin studies suggest that 31% of variance in sociotropy‐related personality (Chen & Li, 2014) and 27% of variance in interpersonal sensitivity (Gillespie et al., 2001) are explained by genetic factors. It is also reported that genetic polymorphism of the brain derived neurotrophic factor (BDNF) is associated with interpersonal sensitivity (Suzuki et al., 2012), while there have been no genetic association studies on sociotropy and autonomy.

As mentioned above, sociotropy/autonomy and interpersonal sensitivity are marked by distinctive interpersonal styles. It is of note that our previous study shows that sociotropy, but not autonomy, is closely related to interpersonal sensitivity (Otani et al., 2012). Furthermore, sociotropy (Otani et al., 2014a) and interpersonal sensitivity (Otani et al., 2014b), but not autonomy (Otani et al., 2014a), are correlated with negative self‐model, an index of attachment insecurity characterized by intimacy and acceptance needs (Bartholomew & Horowitz, 1991). To summarize, sociotropy and interpersonal sensitivity are characterized by a common interpersonal style, that is, a moving toward people style, while autonomy is characterized by a moving away from people style.

The discussions so far made lead to the hypothesis that the A118G OPRM1 polymorphism resulting in altered MOR activity is associated with sociotropy and interpersonal sensitivity, but not autonomy. The purpose of the present study was to test this hypothesis.

## 2 METHODS

Originally, 460 Japanese volunteers were recruited from medical school students and people working at hospitals in Yamagata Prefecture of Japan. A self‐report questionnaire asking the presence of physical or mental diseases and their diagnoses were used to check health condition. Mental health was further checked by a brief interview with six items abstracting from the SCID (First et al., 1997). A1 (In the past month, has there been a period of time when you were feeling depressed or down most of the day) was used for major depressive episode. By the same token, A16, B1, B6, E2, and F68 were used for manic episode, delusions, hallucinations, alcohol abuse, and anxiety disorders, respectively. As a result, five volunteers had physical diseases of moderate to severe degrees, and 14 had mental diseases. In 31 there was a considerable amount of missing data, and in 8 PCR was unsuccessful. The present report dealt with the data of remaining 402 subjects. Two hundred sixty‐three were males, and 139 were females. Their mean age ± SD was 26.0 ± 6.9 years. The Ethics Committee of Yamagata University School of Medicine approved this study, and all subjects gave written informed consent to participate. When obtaining informed consent from the subjects, the information that the refusal or withdrawal of consent does not cause any disadvantage to the subjects were fully provided to them.

The Sociotropy‐Autonomy Scale, a self‐report scale developed by Beck (1983), has the sociotropy and autonomy subscales, each consisting of 30 items. An example from the former is “It is important to be liked and approved of by others,” and an example from the latter is “It is more important to be active and doing things than having close relationships with other people.” The Interpersonal Sensitivity Measure is a 28‐item self‐report scale developed by Boyce and Parker (Boyce & Parker, 1989; Boyce et al., 1998). An example item is “I worry about losing someone close to me.” In the present study, the Japanese versions of the Sociotropy‐Autonomy Scale (Izawa, 1997) and Interpersonal Sensitivity Measure (Kuwabara et al., 1999) with high reliabilities and validities were used. Cronbach's alphas were calculated to assess internal reliability in the present sample, and the values for the sociotropy scale (30 items), autonomy scale (30 items), and Interpersonal Sensitivity Measure (28 items) were 0.89, 0.85, and 0.90, respectively.

DNA was extracted from peripheral blood using a QIAamp DNA Blood Kit (Qiagen, Tokyo, Japan). The A118G polymorphism of the OPRM1 was detected by the PCR method (Noto et al., 2020).

The differences in age and sex distribution among the OPRM1 genotypes were analyzed by one factor analysis of variance and chi‐square test, respectively. Correlations among sociotropy, autonomy, and interpersonal sensitivity were tested by using Pearson correlation analysis. The effects of the OPRM1 polymorphism on scores of sociotropy, autonomy, and interpersonal sensitivity were tested by one factor analysis of covariance with the OPRM1 genotype as a factor and age and sex as covariates, followed by post hoc LDS tests. When the significant difference in personality among the genotypes was found in one factor analysis of covariance, a gene‐dose‐dependent effect on the personality scores was confirmed by using multiple regression analysis where a dependent variable was the personality scores and independent variables were the OPRM1 genotype, age, and sex. In the multiple regression analysis, dummy variables were used for the OPRM1 (A/A = 0, A/G = 1, and G/G = 2) and sex (female = 0 and male = 1). SPSS version 22 (IBM Japan, Tokyo, Japan) was used for statistical analyses. In the statistical analyses for the effects of the OPRM1 polymorphism on three personality (sociotropy, autonomy, and interpersonal sensitivity), Bonferroni's correction for multiple testing was made, that is, multiply a *p* value by three, and a corrected *p* value less than .05 was regarded as significant.

## 3 RESULTS

Scores of sociotropy, autonomy, and interpersonal sensitivity, and the OPRM1 genotype of the subjects are shown in Table 1. There were no significant differences in age (mean age ± SD years; A/A = 25.1 ±4.2, A/G = 26.6 ±8.0, G/G = 26.5 ±8.0; *F* = 2.077, n.s.) and sex distribution (male/female; 99/48 in A/A, 110/68 in A/G, 54/23 in G/G; chi‐square = 2.029, n.s.) among the OPRM1 genotypes. Scores of interpersonal sensitivity were positively correlated with sociotropy scores (*r* = 0.526, *p* < .001) but not with autonomy scores (*r* = 0.075, n.s.), while sociotropy scores were not correlated with autonomy scores (*r* = 0.010, n.s.).

**TABLE 1:** Scores of sociotropy, autonomy, and interpersonal sensitivity, and the OPRM1 genotype of the subjects

Mean age ± SD	26.0 ± 6.9
Male/female, *n*	263/139
Sociotropy, mean ± SD	56.5 ± 13.1
Autonomy, mean ± SD	57.7 ± 10.7
Interpersonal sensitivity, mean ± SD	68.8 ± 12.0
OPRM1 genotype, *n*	
A/A	147
A/G	178
G/G	77

Abbreviation: OPRM1, μ‐opioid receptor gene.

**FIGURE 1:**
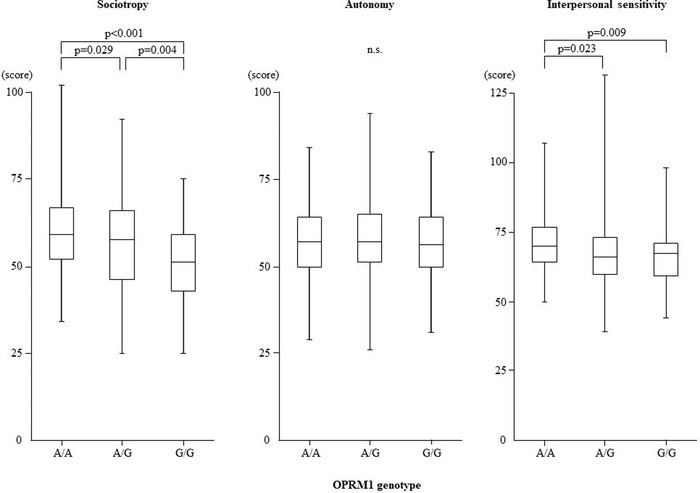
Effects of the OPRM1 genotype on the scores of sociotropy (left) and autonomy (center) evaluated by the Sociotropy‐Autonomy Scale and the scores of interpersonal sensitivity (right) evaluated by the Interpersonal Sensitivity Measure. Differences in the scores of sociotropy, autonomy, and interpersonal sensitivity were tested by analysis of covariance followed by LSD tests. OPRM1, μ‐opioid receptor gene. *p* Values on the figures show the results of post hoc LSD tests

Table 2 shows the results of analyses of covariance for the effects of the OPRM1 genotype on scores of sociotropy, autonomy, and interpersonal sensitivity. There were differences in scores of sociotropy (*F* = 10.182, uncorrected *p* < .001, corrected *p* < .003) and interpersonal sensitivity (*F* = 4.218, uncorrected *p* = .015, corrected *p* = .045), but not autonomy (*F* = 0.549, n.s.), among the A/A, A/G, and G/G genotypes. Post hoc LSD tests showed that sociotropy scores were higher in the A/A group than in the A/G (*p* = .029) and G/G (*p* < .001) groups, and higher in the A/G group than in the G/G group (*p* = .004) (Figure 1). Interpersonal sensitivity scores were higher in the A/A group than in the A/G (*p* = .023) and G/G (*p* = .009) groups (Figure 1).

**TABLE 2:** Results of analyses of covariance for effects of the OPRM1 genotype on scores of sociotropy, autonomy, and interpersonal sensitivity

	OPRM1 genotype					
	A/A	A/G	G/G	*F*	Uncorrected *p*	Corrected *p*
Sociotropy	59.6 ± 11.6	56.4 ± 14.4	50.9 ± 10.6	10.182	<.001	<.003
Autonomy	57.9 ± 10.5	58.0 ± 11.1	56.7 ± 10.6	0.549	.578	n.s.
Interpersonal sensitivity	71.4 ± 10.6	67.8 ± 13.1	66.3 ± 11.2	4.218	.015	.045

*Note*: Figures on the table show mean ± SD except for *F* values. Corrected *p* values were calculated by using Bonferroni's correction for multiple testing.

Abbreviation: OPRM1, μ‐opioid receptor gene.

In the multiple regression analysis, the scores of sociotropy (*R* = 0.272, *β* = −0.219, *p* < .005) and interpersonal sensitivity (*R* = 0.202, *β* = −0.141, *p* = .015) were negatively correlated with the OPRM1 genotype, confirming the gene‐dose‐dependent effects of the OPRM1 genotype on the scores of sociotropy and interpersonal sensitivity.

## 4 DISCUSSION

The A allele of the OPRM1 polymorphism, predictive of increased activity of MOR (Kroslak et al., 2007; Peciña et al., 2015; Weerts et al., 2013; Zhang et al., 2005), was associated with higher scores of sociotropy and interpersonal sensitivity, both of which are highlighted by sensitive and increased interpersonal feelings and behaviors. Especially, the scores of sociotropy and interpersonal sensitivity increased according to the increase of the number of the A allele, that is, in a gene‐dose‐dependent manner. These results are well understood by the A allele's association with increased activity of MOR (Kroslak et al., 2007; Peciña et al., 2015; Weerts et al., 2013; Zhang et al., 2005), which promotes social bonding behaviors (Burkett et al., 2011; Moles et al., 2004; Nummenmaa et al., 2015). As autonomy is featured by self‐schemas focusing on impersonal aspects of life such as task accomplishment and self‐control (Beck, 1983), its lack of an association with the OPRM1 genotype may also be reasonable. Although the present study aimed at elucidating the associations of the OPRM1 polymorphism with depression vulnerabilities, from a broader perspective it provides further evidence for the implication of MOR in social bonding behaviors (Burkett et al., 2011; Machin & Dunbar, 2011; Moles et al., 2004; Nummenmaa et al., 2015).

Our results indicating significant effects of the OPRM1 genotype on sociotropy and interpersonal sensitivity are in line with the results of previous twin studies on these personality traits. Namely, genetic factors accounted for 31% of variation in a group of 22 self‐schemas (Chen & Li, 2014), while they accounted for 27% of variation in interpersonal sensitivity (Gillespie et al., 2001). Incidentally, in the former study (Chen & Li, 2014) self‐schemas were not classified according to their contents. Therefore, the possibility that genetic factors had a strong effect on a cluster of interpersonal schemas (i.e., sociotropic schemas), but a weak or no effect on a cluster of impersonal schemas (i.e., autonomic schemas) cannot be excluded.

As both sociotropy (Hammen et al., 1989; Morse & Robins, 2005) and interpersonal sensitivity (Gao et al., 2017) are shown to be risk factors for depression in longitudinal studies, the previously reported associations of the A allele with depression (Adrian et al., 2015; Kleinjan et al., 2015) or depression‐related events (Hishimoto et al., 2008) may be mediated by the link of this allele with sociotropy and interpersonal sensitivity presented here.

Although our results are likely to be explained by the link of MOR with prosocial behaviors, the connections of MOR with the hypothalamus‐pituitary‐adrenocortical (HPA) axis and BDNF may also be involved and, therefore, worth discussing. It is shown that the endogenous opioid system regulates the response of HPA axis to stress (Bilkei‐Gorzo et al., 2008). In relation to the OPRM1 polymorphism, the A allele is related to greater cortisol response to stress tests such as arithmetic plus public speaking compared to the G allele (Chong et al., 2006). Furthermore, in a study greater cortisol response to interpersonal conflict is associated with higher sociotropy scores (Laurent & Powers, 2006). On the other hand, central administration of endogenous opioids to rats increases mRNA levels of BDNF (Zhang et al., 2006), which is at least partly involved in the formation of interpersonal sensitivity (Suzuki et al., 2012). Therefore, there are possibilities that the HPA axis and BDNF are also involved in our results.

There are a few limitations in this study. First, the present results obtained in Japanese may be difficult to extend to other ethnic groups, since it is reported that a haplotype involving the A118G OPRM1 polymorphism in East‐Asians differs from Europeans (Levran & Kreek, 2020), and that ethnicity‐specific effects of the G allele are found on cortisol response to an opioid blockade (Hernandez‐Avila et al., 2007). Second, the present study was conducted with a cross‐sectional design and, therefore, a longitudinal follow up is needed to confirm if subjects high in the examined personality traits develop depression in the future and actually vulnerable to depression.

In conclusion, the present study suggests that the A118G OPRM1 polymorphism is associated with sociotropy and interpersonal sensitivity, interpersonal vulnerabilities to depression.

## CONFLICT OF INTEREST

The authors declare no conflicts of interest.

## AUTHOR CONTRIBUTIONS

Akihito Suzuki conducted data analysis and prepared the manuscript. Toshinori Shirata, Keisuke Noto, Yoshihiko Matsumoto, Haruka Muraosa, and Mio Abe were responsible for recruiting participants and collecting blood samples. Akihito Suzuki and Yoshihiko Matsumoto detected single nucleotide polymorphism using PCR method. Kaoru Goto was responsible for interpretation of the results. Koichi Otani was the principal investigator of the project and responsible for interpretation of the results. All authors have approved the final manuscript.

### PEER REVIEW

The peer review history for this article is available at https://publons.com/publon/10.1002/brb3.2674

## Data Availability

The data sets generated and/or analyzed during the current study are not publicly available, because the authors are not allowed to publish the raw data of subject's genetic information by the ethics committee of Yamagata University School of Medicine. Data are however available from the corresponding author upon reasonable request.
